# A Study of the Structure of an Anion Exchange Resin with a Quaternary Ammonium Functional Group by Using Infrared Spectroscopy and DFT Calculations

**DOI:** 10.3390/ma17246132

**Published:** 2024-12-15

**Authors:** Katarzyna Chruszcz-Lipska, Elżbieta Szostak

**Affiliations:** 1 Faculty of Drilling, Oil and Gas, AGH University of Krakow, Mickiewicza 30 Ave., 30-059 Kraków, Poland; 2 Faculty of Chemistry, Jagiellonian University in Kraków, Gronostajowa 2 Str., 30-387 Kraków, Poland; szostak@chemia.uj.edu.pl

**Keywords:** anion exchange resin, quaternary ammonium functional group, Amberlite IRA402Cl, IR spectroscopy, DFT calculation, PCM solvation model

## Abstract

The large numbers of ion exchange resins used in various industries (food, pharmaceutitics, mining, hydrometallurgy), and especially in water treatment, are based on cross-linked polystyrene and divinylbenzene copolymers with functional groups capable of ion exchange. Their advantage, which makes them environmentally friendly, is the possibility of their regeneration and reuse. Taking into account the wide application of these materials, styrene–divinylbenzene resin with a quaternary ammonium functional group, Amberlite^®^IRA402, was characterized using a well-known and widely used method, FT-IR spectroscopy. As the infrared spectrum of the tested ion exchange resin was rich in bands, its detailed assignment was supported by quantum chemical calculations (DFT/B3LYP/6-31g** and DFT/PCM/B3LYP/6-31g**). Using appropriate 3D models of the resin structure, the optimization of geometry, the infrared spectrum and atomic charges from an atomic polar tensor (APT) were calculated. A detailed description of the infrared spectrum of Amberlite^®^IRA402 resin (Cl^−^ form) in the spectral range of 4000–700 cm^−1^ was performed for the first time. The charge distribution on individual fragments of the resin structure in aqueous solution was also calculated for the first time. These studies will certainly allow for a better understanding of the styrene–divinylbenzene resin interaction in various processes with other substances, particularly in sorption processes.

## 1. Introduction

The need for continuous development of industries means that the demand for petroleum hydrocarbon (PH) raw materials is constantly growing. It is predicted that by 2030, the consumption of PHs will reach 106.6 million barrels [1,2]. On the other hand, PHs are one of the main pollutants released into the aquatic environment. According to Dadrasnia and Agamuthu, this is about 8.8 million metric tons of crude oil per year [3]. Oil spills float on the surface of water as the density of oil is lower [4]. This may then lead to the evaporation of harmful components of oil, which additionally pollute the ecosystem [5,6]. This is all the more dangerous because the compounds present in crude oil such as aliphatic, aromatic and heterocyclic hydrocarbons, cycloalkanes, saturated acids, asphaltenes, naphthenes, etc. [7,8,9] are most often resistant to biodegradation, have toxic and carcinogenic properties, and also exhibit the ability to bioaccumulate and biomagnify [10,11,12]. Compounds that occur naturally in crude oil include phenols, carbazoles, amides and carboxylic acids, including naphthenic acids, which are a group of polar cycloaliphatic carboxylic acids with 10 to 16 carbon atoms in the molecule [13]. These compounds are a high-priority source of pollution for the aquatic environment because they are toxic to fish [14]. Marsh and Allen have suggested that naphthenic acids in water are resistant to biodegradation [15,16]. Considering the devastating effects of the release of PHs into the environment on human health and other living organisms, it is easy to see that their effective removal is crucial for ecosystem remediation activities [17]. Remediation is a method that can be defined as the process of decontamination and limiting the spread of pollutants in the contaminated environment. This method uses the physical or chemical properties of harmful substances for their removal [18]. In the case of oil spills, the most commonly used technologies are booms, skimmers, solidifiers, dispersants, bioremediation, and in situ burning [19,20]. The problem is that most of these methods are used only to remove the spill without ensuring the reduction in the negative impact of PH components on the environment. Ideally, the remediation method should remove the spill at high speed, be applicable over a large area in difficult marine conditions, enable efficient recovery of PHs, and should not have negative effects on aquatic ecosystems [21]. A promising approach is the combination of traditional spill removal methods with the use of functional materials that can improve the remediation processes. Such materials include hydrophobic and oleophilic polymethylsilsesquioxane or carbon-based aerogels [22,23], nanowire membranes [24], porous boron–nitride [25], modified sponges (e.g., graphene-wrapped sponge) [21,26,27] and ion exchange resins (IERs) [28]. The last of these usually have good stability, long service life, easy regeneration, high adsorption capacity, and can be produced in the form of granules or beads suitable for continuous use [29]. IERs are usually made of a polystyrene–polymer backbone functionalized with various functional groups. The ionized groups on the backbones can absorb contaminants of an ionic character, such as acids present in a solution in dissociated form [30]. Moreover, apart from environmental remediation, IERs have a wide range of other applications, mainly in the pharmaceutical and food processing industries [31]. Their versatility stems from their unique structural and functional properties such as porosity, surface area, and functional group density [32,33,34]. These parameters play a pivotal role in their ion exchange capabilities and are crucial for optimizing performance in specific applications [29]. Naushad and Alothman and Salih et al. linked the functional efficiency of a strongly acidic cation-exchange resin (Amberlite IRA-120) with its structure, demonstrating that the acidic functional groups promote its selectivity and enable rapid removal of toxic Pb^2+^ and Cu^2+^ ions from simulated wastewater [35,36]. Prelot et al. studied the cation-exchange resins Amberlite^®^ IRN77 and Amberlite™ IRN9652 and noted that the individual sorption capacity of these two resins for different cations depends on their cationic charge, size and concentration [37]. These findings led these authors to conclude that the key factor in the selectivity of the decontamination process is the enhancement of the interaction between the used solid sorbent and the selected pollutant species. This can be achieved by surface functionalization of materials with appropriate chemical groups, but this increases the cost of decontamination, leading to the creation of sorbents specific to only one type of contaminant [37]. Kammerer et al. also noted the need for optimization in order to enhance the adsorption and ion exchange processes, which are crucial for the recovery of valuable compounds from food processing by-products [31].

The great advantage of ion exchange resins is their multifunctionality. For example, the strongly basic resin Amberlite IRA-402 has gained attention due to its role in the synthesis of indolizines and pyrrolo [1,2-a]quinolines and isoquinolines, which exhibit significant antibacterial and antifungal activity [38]. This study emphasizes the dual functionality of IRA-402 resin as a catalyst and carrier, showing its potential in medicinal chemistry [38]. Amberlite IRA-402 ion exchange resin has also found an application in the microwave-assisted synthesis of benzoxazole derivatives [39]. In these studies, high anti-inflammatory and antioxidant efficacy of compounds synthesized by this method was demonstrated [39]. The synthesis of biaryl pentacyclic quinolonoquinoxalino-oxazocines in the presence of Amberlite IRA 402(OH) by Priyankar et al. exemplifies the effectiveness of IERs in green chemistry applications [40]. The versatility of ion exchange resins beyond conventional applications is also illustrated by the findings of Jagadeesh Babu et al. and Esteban et al.. They compared and confirmed the efficacy of different ion exchange resins as catalysts for esterification reactions and synthesis of solketal [41,42]. The synthesis of polyoxymethylene dimethyl ethers using macroporous strongly acidic cation-exchange resin (Purolite^®^CT175, Purolite (China) Co., Ltd.) by Wang et al. further exemplifies the effectiveness of IERs in green chemistry applications [43]. Their study highlighted the stability and durability of the resin, which are vital for its repeated use in industrial processes [43]. Marszałek et al. confirmed the possibility of eliminating cadmium from phosphoric acid using PWA5 anionic resin, which is crucial for the production of environmentally friendly fertilizers [44]. Amberlite IRA-402 resins were also studied by Solgy et al. in terms of their adsorption capacity for uranium (VI) ions [45]. The parameters that changed in the system were pH, contact time and adsorbent dosage. The results obtained in the study confirmed the effectiveness of the resin for selective removal of uranium from sulfate solutions, which positions it as a valuable component in environmental remediation activities, in particular in the field of nuclear waste management. The related resin Amberlite IRA-400 Cl− and its hybrids with Mn(OH)_2_ and Cu(OH)_2_ were studied by Mustafa et al. in the context of chromium ion removal from tannery wastes [46]. This time, the absorption was assessed in a system where resin dosage, mixing speed and temperature were varied. The obtained results again indicated the resin’s great potential in industrial wastewater treatment. Additionally, it was found that hybrid ion exchangers have a higher removal capacity and faster kinetics compared to the parent exchanger [46]. The results obtained by Yarahamdi et al. are equally interesting [47] as they proved that Amberlite XAD-7 (AXAD-7) resin impregnated with CYANEX-272 (di-2,4,4-trimethylpentylphosphonic acid) has the ability to remove Ce^3+^ and La^3+^ ions from aqueous solutions. Consequently, this creates the possibility of recovering rare earth elements, which are widely used in the nuclear industry, ceramics and glass production, the petrochemical and catalytic industry, electronics and metallurgy, etc. [48].

Ilalan et al. [49] highlighted the capture capacity of Amberlite^®^IRA resins for absorption of dyes [30,50] and acids, such as simple organic acids with two three or four carbon atoms [51,52,53], phenols and their derivatives [54,55,56], hydroxycarboxylic acids [51,52,53,57] and unsaturated mono- or dicarboxylic acids [49,58].

Despite extensive research on styrene–divinylbenzene resins such as Amberlite^®^IRA-402 and similar resins with different functional groups, there are still significant gaps in knowledge. The sorption mechanism of various chemicals on the resin is not fully understood. This is especially true for petroleum derivatives, which are a source of serious pollution and pose a direct threat to living organisms. Understanding the interaction between ion exchange resins and the type of sorbate under different environmental conditions (water salinity, pH) is crucial for optimizing its application in different water purification contexts. Advanced techniques such as spectroscopic studies and computer simulations are needed to investigate the structure of the sorbent and then understand which changes occur in the resin structure during ion exchange processes. This knowledge can contribute, for example, to the improvement and optimization of water purification/treatment processes and could increase the practical application of environmentally friendly resins under different environmental conditions.

## 2. Materials and Methods

### 2.1. Chemical Reagents

Strongly basic anion exchange resin (type I) AmberLite^®^IRA402 (Cl form) (CAS number: 52439-77-7) was purchased from Thermo Scientific (Illkirch-Graffenstaden, France). It is a styrene–divinylbenzene resin with a quaternary trimethylammonium functional group in a gel form. Pale yellow beads of resin have a diameter of ca. 0.7 mm and moisture holding capacity of ca. 52%. AmberLite^®^IRA402 was taken for measurements of the FT-IR spectrum without any preparation after opening the manufacturer’s original packaging.

### 2.2. Infrared Spectroscopy

Measurement of the infrared absorption spectrum of AmberLite^®^IRA402 was performed with an Avatar 360 FT-IR spectrometer (Thermo Nicolet, Thermo Fisher Scientific, Waltham, MA, USA) with a deuterated triglycine sulfate (DTGS) detector. The spectrum was recorded under room conditions in the spectral range of 4000–400 cm^−1^. The KBr pellet method with transmission mode was used. A mixture of 150 mg KBr (for IR spectroscopy, Thermo Scientific) and 2 mg of AmberLite^®^IRA402 was ground in an agate mortar. Potassium bromide was previously dried in a muffle furnace for 24 h at 500 °C before use. The KBr pellets were prepared on a 2−ton mini pellet press (Specac Ltd. Company, Orpington, UK) by applying 1.9 tons of pressure for 3 min. The spectrum was performed in duplicate with 256 scans and a resolution of 2 cm^−1^.

### 2.3. DFT Calculations

**Figure 1:**
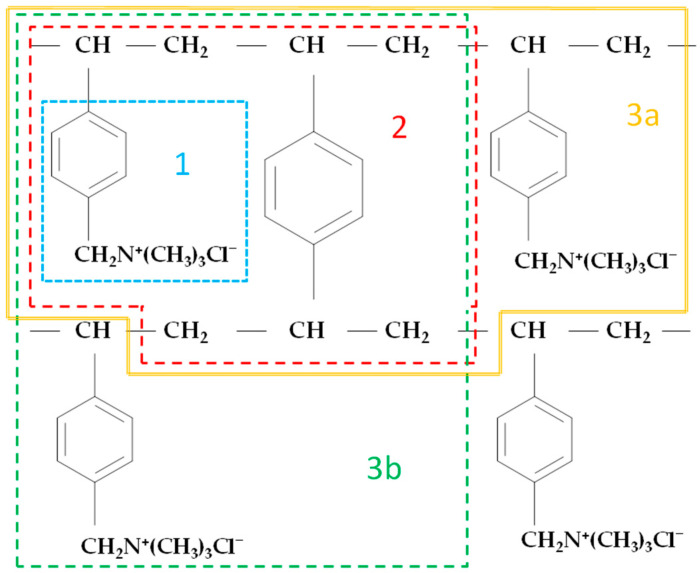
Fragment of the structure of the styrene–divinylbenzene anion exchange resin with a trimethylammonium functional group. Names of models reflecting the structure of the resin: 1, 2, 3a and 3b.

All calculations were performed using the Gaussian 16 program packages [59]. These calculations were carried out at the DFT level, with the most commonly used B3LYP functional and a 6-31g** basis set [60,61]. Since ion exchange resins are most often used for separation, purification and treatment processes in aqueous solutions, all calculations for the studied styrene–divinylbenzene resin were performed in two ways. Computer simulations of the models of the resin structure were performed for the isolated form and for the PCM (polarizable continuum model) solvation model. If the processes take place in different types of solutions, the solvent model calculations allow for better description of the experimental conditions that prevail there. In the PCM method, the solvent model is implemented in such a way that the solute (in our case, the appropriate resin structure model) occupies a cavity with a solvent field characterized by its individual dielectric constant (in the case of water, this constant is 78.4) [62,63]. In the first step, different fragments of Amberlite IRA402 anion exchange resin’s structure were defined as models. The model structures created always contained the benzyltrimethylammonium cation (functionalized group of IRA402). These four models reflected the resin structure (Figure 1) in cationic form and contained the following numbers of atoms, charge and multiplicity, respectively: 27, +1, 1 (model 1); 70, +1, 1 (model 2); 101, +2, 1 (model 3a) and 101, +2, 1 (model 3b). The geometries of the structures were fully optimized with no restriction. In the second step, the infrared frequencies were calculated. No imaginary frequencies were determined for any of the optimized structures. Theoretical IR spectra of the model structures of the anion exchange resin Amberlite IRA402 were obtained by representing each band as a Lorentzian-shaped curve, and half bandwidths of 4 cm^−1^ were used to take temperature broadening into account. The calculated harmonic frequencies were not scaled. The assignment of individual experimental bands to the corresponding modes in the theoretical spectra of IRA 402 resin in the range of 4000–700 cm^−1^ was based on a direct comparison of the experimental and calculated spectra that took into account the frequency sequence and intensity pattern visualized using GaussView 5.0 [64].

The output files from the Gaussian program for the infrared frequency calculations also contain the data of the charge distribution on all individual atoms in the structure. The APT (atomic polar tensor) method is one of the most recognized and widely used methods for assigning an electronic charge to an individual atom [65]. In this approximation, the atomic charge is related to the trace of the corresponding atomic polar tensor, i.e., the tensor of the derivatives of the dipole moment with respect to atomic Cartesian coordinates. Data on the value and sign of the charge on a specific atom assist in the recognition of the structure of a given chemical compound and, consequently, help understand the physical and chemical properties of the whole material. This is important, for example, for predicting the sorption processes of ion exchange resins.

## 3. Results and Discussion

### 3.1. Geometry of Models of the Structure of the Styrene–Divinylbenzene Resin with Trimethylammonium Functional Group

**Figure 2:**
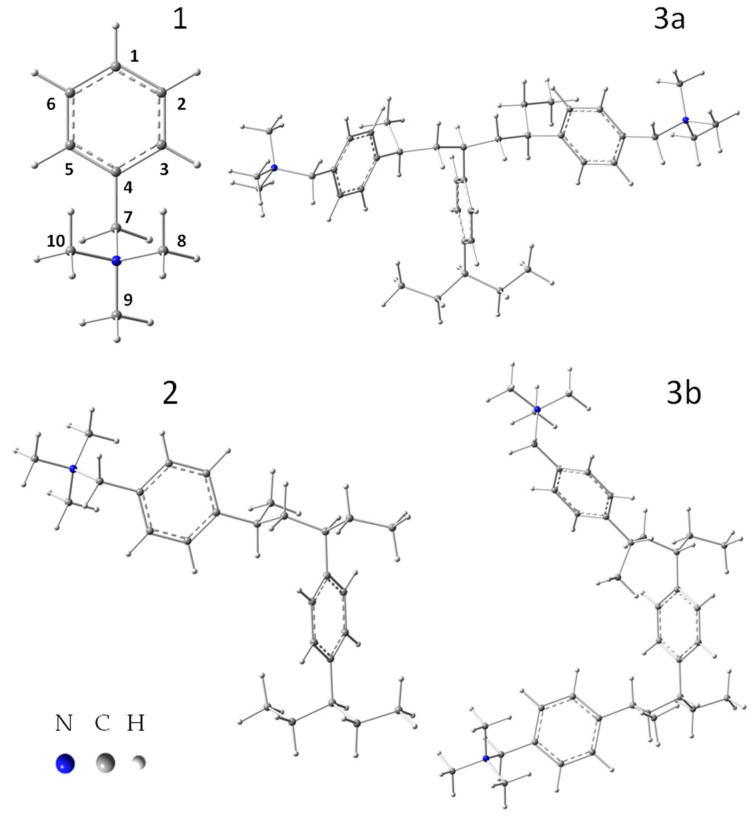
Optimized geometry for the models (1, 2, 3a and 3b) of the structure of the styrene–divinylbenzene anion exchange resin with a quaternary ammonium functional group (Amberlite^®^IRA402) (DFT/PCM/B3LYP/6-31g**).

When analyzing the structure of the styrene–divinylbenzene resin with a trimethylammonium functional group such as Amberlite^®^IRA402Cl (Figure 1) [66,67,68], four models in cationic form were considered in this work. For these models, the geometries were optimized and then the IR spectra and the charge distribution on all individual atoms were calculated. The first, smallest model considered was the benzyltrimethylammonium cation (model 1, Figure 1 and Figure 2). The second model (model 2) contained an additional benzene ring, which is a cross-linking element in the resin structure; the subsequent models 3a and 3b also include additional benzyltrimethylammonium cations (Figure 1). Models 2, 3a and 3b (Figure 2) were created as shown in Figure 1 (the colored line limits the size of each model). Since the models were created by “cutting out” fragments of the polymer structure of the resin, additional methyl groups were added at each “cut” of the resin structure (compare Figure 1 and Figure 2). Geometry optimization for these all structures was performed in two ways: for the isolated structure (DFT/B3LYP/6-31g**) and for the structure where the solvation effect of water was taken into account (DFT/PCM/B3LYP/6-31g**). The optimized geometries of the four investigated models of the structure of the studied resin calculated at the DFT/PCM/B3LYP/6-31g** level of theory are presented in Figure 2.

As is shown in Table 1, the calculated bond distances between atoms in the benzyltrimethylammonium cation that contains the functional group of the resin led to similar results for all models. The greatest similarity was shown for the bond lengths calculated for models 3a and 3b. On the other hand, the greatest differences can be observed between the calculations for the isolated structure and the calculations for the PCM solvation model for models 1 and 2. Selected crystallographic data for the benzyltrimethylammonium cation (crystal structure with rac-1,1-Bi-2-naphtholand [69] and tetrachloroferrate (III) [70]) are gathered in Table 1 and are in good agreement with the experiment.

### 3.2. Comparison of the Theoretical and Experimental Spectra of the Styrene–Divinylbenzene Resin with a Trimethylammonium Functional Group (IRA402)

The literature data indicate that infrared spectroscopy has already been used to characterize the styrene–divinylbenzene resin Amberlite^®^IRA 402 [30,71,72,73,74,75]. In these works, the authors use IR spectroscopy to confirm that the resin surface was modified or to characterize possible interactions between the IRA 402 resin and sorbate in the sorption process. However, the authors mention only a few main bands in the infrared spectrum to describe the issue under discussion. To our knowledge, the infrared spectrum of styrene–divinylbenzene resin with a quaternary amine group has not been described so far in as much detail as is presented in this work. The knowledge gained from understanding the relationship between the band at a given wavenumber and the vibrations of the corresponding fragment of the material structure enables, thanks to IR spectroscopy, a deeper and better description of the interactions of this material with other chemical compounds during various processes.

**Figure 3:**
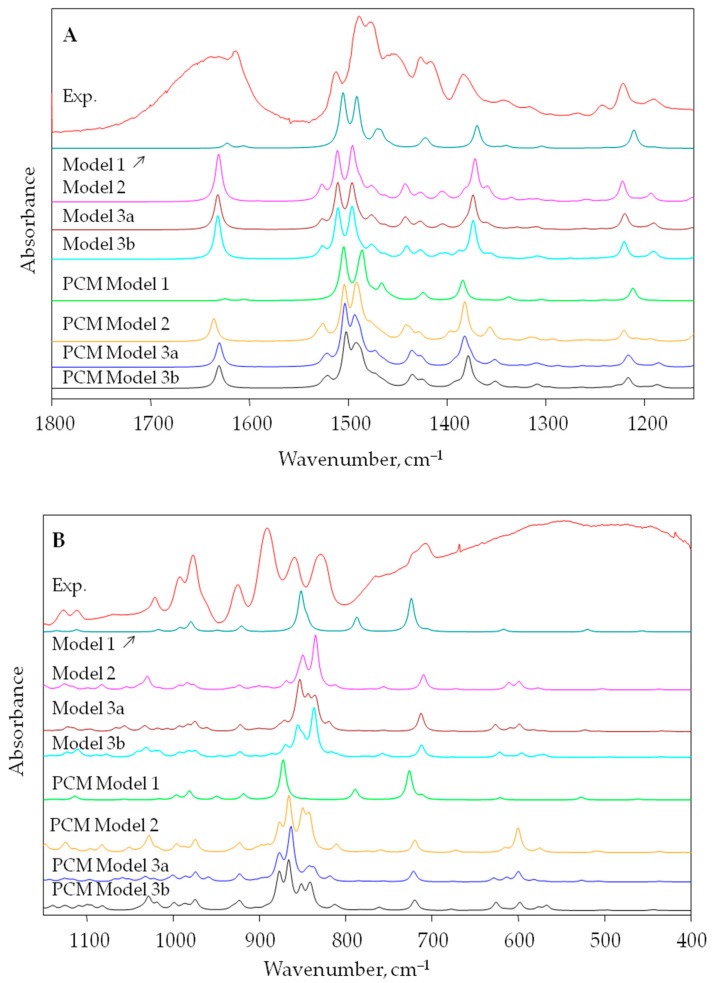
Comparison of the experimental IR spectrum of IRA402 exchange resin with theoretical IR spectra (B3LYP/6-31g**) obtained for optimized model structures 1–3 (Figure 2) in two spectral ranges: 1800–1150 cm^−1^ (**A**) and 1150–400 cm^−1^ (**B**). Calculations for the isolated structures—models 1, 2, 3a and 3b; calculations for structures in water (PCM solvation model)—PCM models 1, 2, 3a and 3b.

A comparison of the theoretical spectra obtained for four different models reflecting the structure of the IRA402 resin and its experimental spectrum in the range of 1800–1150 and 1150–400 cm^−1^ is shown in Figure 3. For the wavenumber range 1800–1150 cm^−1^ (Figure 3A), the theoretical spectra for both isolated structures and in the water environment (PCM model) for models 2 and 3a and 3b are very similar and provide a very good fit to the experimental data. The order and shape of the bands in the spectrum are very similar; however, the calculated frequency values are shifted towards higher wavenumbers, which is a result of calculation error (inaccurate description of the electron–electron interaction; the neglect of anharmonicity). In the second wavenumber range 1150–400 cm^−1^ (Figure 3B), the calculated spectra differ much more and their fit to the experimental data is not as good as in the previously discussed spectral range. This is due not only to the limitation of the applied calculation method but also the limitation of the resin structure model used. A more extended model which contains a larger fragment of the polymer structure could probably better reflect the real structure of this polymer material.

**Figure 4:**
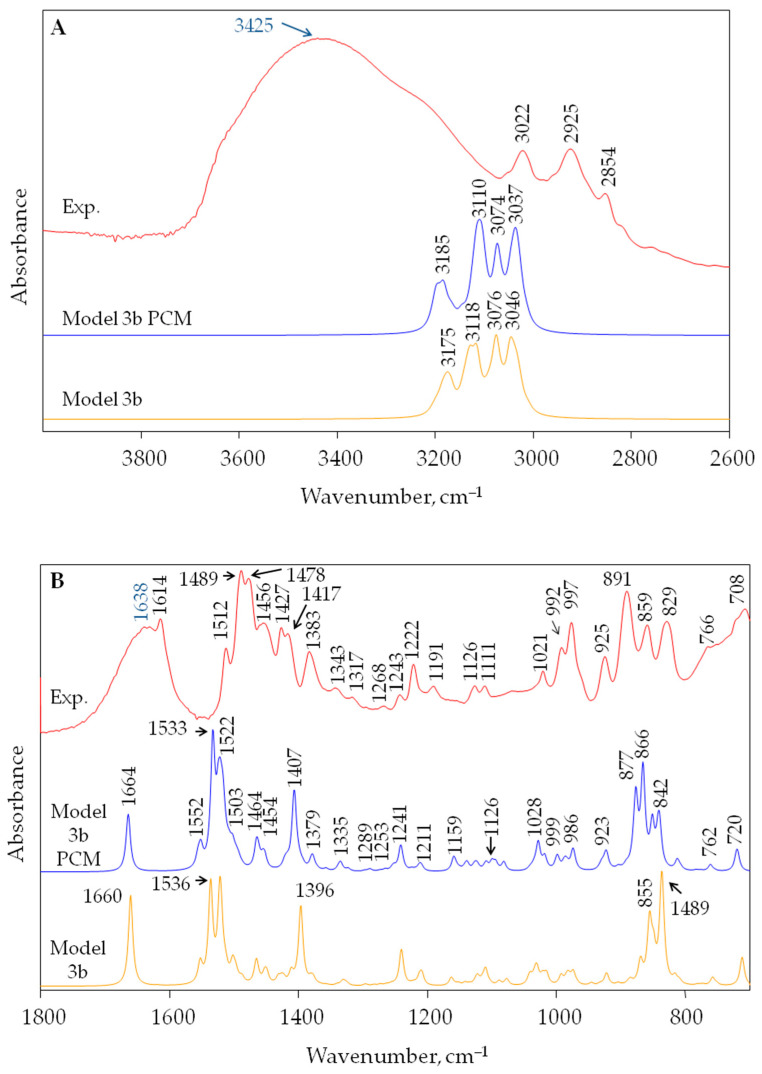
Comparison of the experimental IR spectrum of Amberlite^®^IRA402 exchange resin with the theoretical IR spectra (B3LYP/6-31g**) obtained for model 3b (Figure 2) in two spectral ranges: 4000–1800 cm^−1^ (**A**) and 1800–700 cm^−1^ (**B**). Calculations for the isolated structure 3b—blue line; calculations for structure 3b in water (PCM solvation model)—orange line.

The best fit to the experiment in the whole studied wavenumber range can be seen for model 3b (Figure 3), which contains not only two benzyltrimethylammonium cations but also a benzene molecule which is a cross-linking element in the studied resin structure. The best reproduction of the experiment seems to be for the solvation PCM model for structure 3b (Figure 3). IRA402 resin is a highly hygroscopic compound and water molecules are immediately absorbed by its structure, as is reflected in the IR spectrum. In the IR spectrum of Amberlite^®^IRA402, broad and intense IR bands at 3425 and 1638 cm^−1^ are observed that are related to the stretching and bending vibrations of the O-H groups in water molecules, respectively (Figure 4).

Taking into account the best fit of the calculated spectrum to the experimental one, the detailed assignment of bands to the vibrations of the corresponding fragments of the resin structure was performed on the basis of calculations for model structure 3b (PCM solvation model) (Figure 4 and Table 2). Additionally, for greater clarity, the vibrational modes with atomic displacements localized mainly in the benzene rings were compared with those for the neutral benzene; these are presented in Wilson notation in Table 2 [76,77].

As can be seen in Figure 4, the most intense bands in the fingerprint region are observed at 1489 and 1478 cm^−1^; according to the calculations (their analogs are at 1533 and 1522 cm^−1^, Table 2), they are related to bending vibrations in methyl groups attached to the nitrogen atom. The same modes are also observed at 1491, and 1480 cm^−1^ for the experimental and calculated (1513 and 1502 cm^−1^, respectively) spectra of 4-(trimethylammonium) benzoic acid chloride [78]. In the case of the vibration at 1478 cm^−1^ (similar to vibration at 1456 cm^−1^) a scissoring vibration of the –CH_2_– group bound to the N atom is observed (Table 2). Other experimentally highly active IR absorbance bands related to the vibrations of the –CH_2_–N(CH_3_)_3_ resin functional group, which may undergo greater changes upon interaction, especially with anionic reagents, are the bands at 1417, 1383, 1222, 992, 891, 829 and 708 cm^−1^. The band at 1417 cm^−1^ is associated with the symmetric bend for methyl groups bound to the nitrogen atom (an umbrella bend vibration). The origin of this band was also confirmed by DFT calculations performed for (trimethylammonium) benzoate iodide and 4-(trimethylammonium)benzoic acid chloride [78,79]. The IR signal at 1383 cm^−1^ (calculated at 1407 cm^−1^) is caused by the wagging vibrations of the –CH_2_– group, which is located between the benzene ring and the –N(CH_3_)_3_) group. The band at 992 cm^−1^ (calculated 999 cm^−1^) is related to the rocking vibration of the same –CH_2_– group. The infrared signals at 1222 and 891 cm^−1^ originate from the C–C and C–N stretching vibrations, respectively. The bands at 829 and 708 cm^−1^ are due to the mixed deformation of the –N(CH_3_)_3_ group and C–H out-of-plane bending vibration (Table 2). It is worth emphasizing here that the nature and presence of all the above-mentioned vibrations were also postulated by Mączka et al. based on FT-IR analysis performed for benzyltrimethylammonium cations (BeTriMe^+^) [80]. Lower-intensity infrared signals related to the vibrations of the resin functional group –CH_2_–N(CH_3_)_3_ occur at 1343, 1268 and 1243 cm^−1^. The presence of these vibrations was also predicted by Mączka’s DFT spectra calculations [80]; however, with the exception of the mode at ca. 1240 cm^−1^, these bands were not observed in the experimental infrared spectrum of (BeTriMe^+^).

The infrared spectrum of the IRA402 resin also shows intense bands not related to the quaternary amine functional group. These are bands closely related to the resin skeleton and are at 1614, 1512, 1427, 997, 925 and 859 cm^−1^. The IR signal in this spectrum at 1614 cm^−1^ is due to the C=C stretching vibration in the benzene ring. The corresponding band in the calculated spectrum is found at 1664 cm^−1^ (Figure 4 and Table 2). The band at 1512 cm^−1^ (calculated at 1521 cm^−1^) originates from the in-plane deformation vibration of the C–H group in the benzene ring (mainly cross-linked). The presence of these two bands was also predicted by Baldea for the spectra of neutral benzene; however, the calculated frequencies were slightly higher than in this work [77]. The origin of the band at 1427 cm^−1^ is more complex (Figure 4, Table 2). It is connected to in-plane deformation vibration of the C–H groups in the benzene ring, the C–C stretching vibration in rings and C–H bending, where the C atom is an aliphatic carbon atom (CH) directly bound to the ring. The reflection of the above-mentioned combination of vibrations can be found in the spectra of benzene and benzyltrimethylammonium cations in [77,80]. The intense infrared signal at 997 cm^−1^ originates from out-of-plane deformation vibration of the C–H groups in the benzene ring. The similar origin has a band at 859 cm^−1^ (calculated signal at 866 cm^−1^). However, this band is not only due to in-plane deformation vibration of the C–H group in the benzene ring but also due to the rings’ deformation vibrations. Similar bands were reported by Baldea and Szafran in [77,78]. The band at 925 cm^−1^ (calculated signal at 923 cm^−1^) is connected mainly to the rocking vibration of C–H in –CH_2_– groups in the aliphatic carbon chain between the benzene rings. A band of a similar nature was recorded at 988 cm^−1^ for benzyltrimethylammonium cations in [80]. Lower-intensity IR signals in the spectral range of 1800–700 cm^−1^ related to resin skeleton vibration are observed at 1317, 1191 and 1021 cm^−1^. Their calculated counterparts are at 1335, 1211 and 1028 cm^−1^, respectively. The origin of these bands is explained in detail in Table 2 and corresponds with the results presented in [76,77].

Moreover, in the spectral range of 4000–1800 cm^−1^, calculations predict the occurrence of bands with a maximum at 3185, 3110, 3074 and 3037 cm^−1^. These bands are formed by the overlapping of many theoretical vibrational modes. Theoretical modes at a high wavenumber with a maximum at 3185 cm^−1^ are related to the C–H stretching vibrations in the –CH_2_–N(CH_3_)_3_ functional group of the resin (both in the –CH_3_ and –CH_2_– groups), as well as the C–H stretching vibrations in the aromatic rings. They were also registered for benzene, 4-(trimethylammonium) benzoate iodide, 4-(trimethylammonium) benzoic acid chloride and benzyltrimethylammonium cations in [77,80]. The rest of the vibrational modes are, according to the calculations, connected to the C–H stretching vibration in aliphatic chains between the aromatic rings. In the experimental spectrum, three intense bands with a maximum at 3022, 2925 and 2854 cm^−1^ are observed in this wavenumber region.

As expected, the calculations showed that the bands associated with the vibrations of the amino group in the resin structure are the most sensitive to the introduction of water into the environment of the modeled structure (using the PCM model) (Figure 4). The largest changes in the spectra calculated for the isolated structure and for the PCM model are noticeable in the frequency range of 877–842 cm^−1^ (Figure 4). The strong bands at 1533 and 1407 cm^−1^ are also sensitive to the applied calculation procedure. For the isolated structure, their counterparts are 1535 and 1397 cm^−1^. The calculated strong band that is sensitive to the change in the model environment is also the C=C stretching vibration, which occurs for the isolated structure of model 3b at 1660 cm^−1^. This band appears at 1664 cm^−1^ using the solvation model and is much less intense.

### 3.3. Distribution of APT Atomic Charges on the Benzyltrimethylammonium Cation

**Figure 5:**
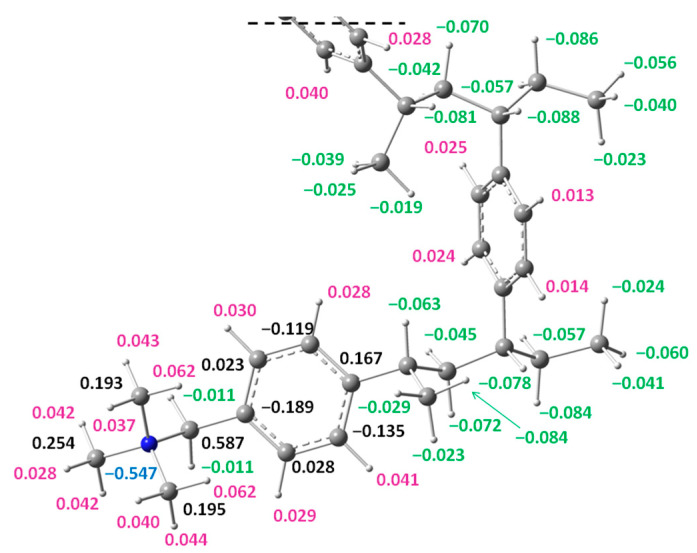
Distribution of atomic charges (APT) on individual atoms for the functional group of the resin and its surroundings (Figure 2, model 3b, DFT/PCM/B3LYP/6-31g**).

Understanding the charge distribution in the resin structure allows us to predict the possibility of its interaction with other substances. The atomic charge distribution (APT) on selected atoms for the models of the resin structure of IRA402 (models 1–3) is presented in Table 3 and for model 3b in Figure 5. The calculated values are similar for all models. The greatest discrepancies can be observed for model 1 in relation to the more extensive models (models 2, 3a and 3b). Differences are also observed when the solvent model is taken into account. For the same theoretical model in the presence of water (PCM), the atomic charge values for carbon atoms increase, while for hydrogen atoms, they decrease slightly. It can be observed (Table 3 and Figure 5) that both carbon and hydrogen atoms in the –CH_3_ groups attached to the nitrogen atom carry a positive charge (calculation for all models). There is also a positive charge on the hydrogen atoms of aromatic rings. Hydrogen atoms attached to the remaining aliphatic carbon atoms in the resin structure are always negatively charged. Thus, the theoretical calculation confirmed that in the case of the tested IRA402 resin, the –N(CH_3_)_3_ group is the site with the highest positive charge in the whole structure and can play a key role in the ion exchange process.

## 4. Conclusions

In this work, a study of the structure of styrene–divinylbenzene resin with the quaternary ammonium functional group Amberlite^®^IRA402 (in Cl^−^ form) was performed. The resin was characterized by using FT-IR spectroscopy and density functional theory calculations. The calculations (geometry optimization, infrared frequencies and the atomic charge from the corresponding atomic polar tensor) were performed for four models of the resin structure with different atom numbers. Taking into account that ion exchange resins are used in various processes (especially sorption) that usually occur in an aqueous environment, the calculations were carried out for isolated structures (DFT/B3LYP/6-31g**) and for a model taking into account the aqueous solution (DFT/PCM/B3LYP/6-31g**).

As a result of the conducted studies, individual bands in the complex IR spectrum of Amberlite^®^IRA402 in the spectral range of 4000–700 cm^−1^ were assigned to vibrations of individual fragments of the resin structure. This detailed assignment of bands can be used in other studies (using vibrational spectroscopy) to better understand the chemical properties of styrene–divinylbenzene resin as a widely used industrial material with various applications. In particular, in the field, the sorption processes of petroleum substances from waters are of urgent interest.

The paper also presents, for the first time, the charge distribution on individual atoms in the resin structure. The calculations confirmed that the –N(CH_3_)_3_ group is the place with the largest positive charge in the entire structure. The positive charge is also located on the hydrogen atoms of aromatic rings, and the hydrogen atoms of aliphatic carbon atoms that form the resin skeleton are negatively charged.

Certainly, further comparative studies with similar resins would be of great interest in the future. According to our knowledge, the detailed assignment of infrared absorption bands to the vibrations of individual fragments of structures of other styrene–divinylbenzene resins has not yet been described in the literature, which constitutes a significant gap in the knowledge on this issue. It also seems interesting to determine the charge distribution on individual atoms for the different functional groups of the resins by using quantum–chemical calculations. These studies could demonstrate how the presence of other, even slightly different, functional groups (e.g., trimethylammonium, triethylammonium) in the resin structure influences the charge distribution and affects, for example, the sorption efficiency of various compounds.

## Figures and Tables

**Table 1:** Comparison of experimental [69,70] and calculated geometrical parameters (selected bond distances and angles) for benzyltrimethylammonium cation. Calculations depend on the adopted model and its surroundings (isolated structure—DFT/B3LYP/6-31g**, structure in water solution DFT/PCM/B3LYP/6-31g**).

Geometrical Parameters * [Å, °]	Crystallographic Data		Calculations							
			Model 1		Model 2		Model 3a		Model 3b	
	[69]	[70]	Isolated	PCM	Isolated	PCM	Isolated	PCM	Isolated	PCM
C_1_–C_6_ C_1_–C_2_	1.387 1.381	1.374 1.360	1.396 1.396	1.396 1.396	1.405 1.403	1.404 1.401	1.405 1.405 1.403 1.403	1.404 1.404 1.401 1.402	1.405 1.405 1.402 1.402	1.404 1.404 1.401 1.402
C_6_–C_5_ C_2_–C_3_	1.393 1.389	1.389 1.370	1.394 1.394	1.395 1.395	1.391 1.392	1.392 1.394	1.393 1.393 1.392 1.392	1.393 1.393 1.394 1.394	1.393 1.391 1.394 1.391	1.393 1.393 1.394 1.394
C_5_–C_4_ C_3_–C_4_	1.397 1.390	1.391 1.383	1.404 1.404	1.403 1.403	1.405 1.404	1.404 1.402	1.405 1.405 1.403 1.403	1.404 1.404 1.402 1.402	1.405 1.405 1.403 1.403	1.404 1.404 1.402 1.403
C_4_–C_7_	1.504	1.488	1.502	1.506	1.498	1.505	1.501 1.501	1.505 1.505	1.501 1.501	1.504 1.505
C_7_–N	1.524	1.520	1.558	1.546	1.562	1.548	1.559 1.559	1.548 1.548	1.559 1.560	1.548 1.548
N–C_8_ N–C_9_ N–C_10_	1.502 1.502 1.496	1.491 1.488 1.495	1.505 1.506 1.505	1.505 1.507 1.505	1.504 1.505 1.505	1.505 1.507 1.505	1.505 1.505 1.507 1.507 1.505 1.505	1.505 1.505 1.507 1.507 1.505 1.505	1.505 1.505 1.506 1.506 1.505 1.504	1.505 1.505 1.507 1.507 1.505 1.505
C_4_–C_7_–N	115.63	115.38	115.58	115.67	115.49	115.61	115.70 115.69	115.67 115.58	115.82 115.71	115.63 115.62
C_7_–N–C_9_ C_7_–N–C_8_ C_7_–N–C_10_	111.44 107.30 110.66	110.55 108.52 109.75	107.92 110.70 110.70	107.51 110.99 110.98	108.01 110.57 110.57	107.70 110.99 110.76	107.95 107.95 110.67 110.67 110.69 110.66	107.58 107.61 110.90 110.89 110.90 109.87	107.97 107.96 110.70 110.66 110.53 110.64	107.62 107.66 110.86 110.93 110.93 110.83
C_10_–N–C_8_ C_8_–N–C_9_	109.76 108.55	110.71 108.89	109.21 109.14	109.11 109.10	109.23 109.22	109.31 108.95	109.23 109.24 109.14 109.12	109.24 109.30 109.06 109.06	109.26 109.25 109.12 109.13	109.19 109.31 109.14 109.02

* Atom numbering according to Figure 2.

**Table 2:** Assignment of experimental infrared bands on the basis of DFT/PCM/B3LYP/6-31g** calculations for the styrene–divinylbenzene resin with a trimethylamonium functional group (model 3b of Amberlite^®^IRA-402Cl, Figure 2).

Experiment	Calculations	Mode ^a^	Assignment
3022 2925 2854	3185	2 + 20a + 20b	ν C–H in –CH_3_ groups (bound to N), ν C–H in rings, ν C–H in –CH_2_– (bound to N)
	3110		ν C–H (C atom is an aliphatic carbon atoms between rings)
	3074		
	3037		
1614	1664	8a + 8b	ν C=C rings
1512	1552	19b	δ C–H in-plane in ring (mainly cross-linked)
1489	1533		δ_as_ C–H in –CH_3_ groups (bound to N)
1478	1522		δ_as_ C–H in –CH_3_ groups (bound to N) δ_scissoring_ –CH_2_– (bound to N)
1456	1503		δ_as_ C–H in –CH_3_ groups (bound to N) δ_scissoring_ –CH_2_– (bound to N)
1427	1464	19b	δ C–H in-plane in rings, ν C–C in rings, δ C–H (C atom is an aliphatic carbon atom directly bound to ring)
1417	1454		δ_umbrella_ –CH_3_ groups (bound to N)
1383 1373 sh	1407 1405		δ_wagging_ –CH_2_– (between ring and –N(CH_3_)_3_) δ C–H and ν C–C (C atom is an aliphatic carbon atom directly bound to cross-linked benzene ring)
1343	1379		δ _twisting_ C–H in –CH_2_– (between ring and –N(CH_3_)_3_)
1317	1335	14	δ _twisting_ C–H in –CH_2_– in aliphatic chain between rings, δ C–H in-plane in rings
1268	1289		ν C–N, δ C–H in aliphatic chain between rings
1243	1253		δ_wagging_ C–H in –CH_2_– (between ring and –N(CH_3_)_3_), δ C–H in –CH_3_ groups (bound to N)
1222	1241	9a	ν C–C between C_ring_ and –CH_2_–N(CH_3_)_3_, δ C–C in ring
1191	1211	9b	δ C–C in ring, δ C–H in-plane in ring, ν C–C between C_ring_ and C in aliphatic chain between rings
1126	1159 1140 1126	9b 15 18a	δ C–H in-plane in ring
1111	1109 1100		ν C–C in aliphatic chain, δ C–H in aliphatic chain
1021	1028	12	δ ring, ν C–C in aliphatic chain, δ C–H in aliphatic chain
992	999		δ _rocking_ C–H in –CH_2_– (between ring and –N(CH_3_)_3_)_,_
977 960 sh	986 974	5	γ C–H out-of-plane in ring
925	923		δ _rocking_ C–H in –CH_2_– in aliphatic chain between rings
891	877	11	ν C_CH2_–N, γ C–H out-of-plane in ring, δ ring
859	866	10b	γ C–H out-of-plane in ring, δ ring
829	851 842	10b	γ C–H out-of-plane in ring, δ –N(CH_3_)_3_
766	762	4	δ –N(CH_3_)_3_, γ C–H out-of-plane in ring
708	720	4	δ –N(CH_3_)_3_, γ C–H out-of-plane in ring

^a^ Mode in Wilson notation.

**Table 3:** Selected APT charges for benzyltrimethylammonium cation, depending on the adopted model (1–3) and its surroundings (isolated structure—DFT/B3LYP/6-31g**; structure in water solution—DFT/PCM/B3LYP/6-31g**).

Atom Number *	Calculations							
	Model 1		Model 2		Model 3a		Model 3b	
	Isolated	PCM	Isolated	PCM	Isolated	PCM	Isolated	PCM
C_7_	0.479	0.535	0.578	0.586	0.562 0.560	0.588 0.588	0.564 0.565	0.587 0.588
C_8_	0.146	0.195	0.140	0.194	0.143 0.141	0.194 0.194	0.143 0.144	0.193 0.195
C_9_	0.205	0.249	0.212	0.253	0.212 0.213	0.253 0.254	0.216 0.214	0.254 0.254
C_10_	0.146	0.195	0.143	0.194	0.140 0.144	0.193 0.193	0.140 0.141	0.195 0.193
N	−0.447	−0.526	−0.488	−0.547	−0.481 −0.481	−0.547 −0.547	−0.483 −0.483	−0.547 −0.547
H_C7_	−0.001 −0.001	−0.004 −0.004	−0.008 −0.010	−0.008 −0.011	−0.007 −0.007 −0.009 −0.009	−0.010 −0.009 −0.010 −0.010	−0.007 −0.006 −0.008 −0.008	−0.011 −0.011 −0.011 −0.011
H_C8_	0.066 0.047 0.042	0.061 0.044 0.042	0.068 0.048 0.038	0.062 0.044 0.038	0.066 0.065 0.049 0.049 0.041 0.042	0.063 0.063 0.043 0.043 0.038 0.039	0.064 0.065 0.048 0.047 0.041 0.039	0.062 0.062 0.044 0.044 0.040 0.040
H_C9_	0.048 0.048 0.035	0.042 0.042 0.029	0.048 0.048 0.029	0.043 0.040 0.027	0.049 0.049 0.049 0.049 0.033 0.035	0.043 0.042 0.041 0.041 0.027 0.027	0.050 0.051 0.050 0.049 0.033 0.033	0.042 0.041 0.042 0.040 0.028 0.028
H_C10_	0.066 0.047 0.042	0.061 0.044 0.042	0.068 0.047 0.037	0.063 0.044 0.040	0.066 0.049 0.042	0.063 0.062 0.044 0.043 0.040 0.038	0.065 0.066 0.049 0.051 0.041 0.041	0.062 0.062 0.043 0.043 0.037 0.037

* Atom numbering taken from Figure 2.

## Data Availability

The original contributions presented in the study are included in the article; further inquiries can be directed to the corresponding author.
